# Role of the endocannabinoid system in obesity induced by neuropeptide Y overexpression in noradrenergic neurons

**DOI:** 10.1038/nutd.2015.1

**Published:** 2015-04-27

**Authors:** L H Vähätalo, S T Ruohonen, S Mäkelä, L Ailanen, A-M Penttinen, T Stormi, T Kauko, F Piscitelli, C Silvestri, E Savontaus, V Di Marzo

**Affiliations:** 1 Department of Pharmacology, Drug Development and Therapeutics and Turku Center for Disease Modeling, University of Turku, Turku, Finland; 2 Drug Research Doctoral Program, University of Turku, Turku, Finland; 3 Department of Biostatistics, University of Turku, Turku, Finland; 4 Endocannabinoid Research Group, Institute of Biomolecular Chemistry, Consiglio Nazionale delle Ricerche, Pozzuoli (NA), Italy; 5 Unit of Clinical Pharmacology, Turku University Hospital, Turku, Finland

## Abstract

**Objective:**

Endocannabinoids and neuropeptide Y (NPY) promote energy storage via central and peripheral mechanisms. In the hypothalamus, the two systems were suggested to interact. To investigate such interplay also in non-hypothalamic tissues, we evaluated endocannabinoid levels in obese OE-NPY^DβH^ mice, which overexpress NPY in the noradrenergic neurons in the sympathetic nervous system and the brain.

**Methods:**

The levels of the endocannabinoids anandamide and 2-arachidonoylglycerol (2-AG) were measured in key regulatory tissues, that is, hypothalamus, pancreas, epididymal white adipose tissue (WAT), liver and soleus muscle, over the development of metabolic dysfunctions in OE-NPY^DβH^ mice. The effects of a 5-week treatment with the CB1 receptor inverse agonist AM251 on adiposity and glucose metabolism were studied.

**Results:**

2-AG levels were increased in the hypothalamus and epididymal WAT of pre-obese and obese OE-NPY^DβH^ mice. Anandamide levels in adipose tissue and pancreas were increased at 4 months concomitantly with higher fat mass and impaired glucose tolerance. CB1 receptor blockage reduced body weight gain and glucose intolerance in OE-NPY^DβH^ to the level of vehicle-treated wild-type mice.

**Conclusions:**

Altered endocannabinoid tone may underlie some of the metabolic dysfunctions in OE-NPY^DβH^ mice, which can be attenuated with CB1 inverse agonism suggesting interactions between endocannabinoids and NPY also in the periphery. CB1 receptors may offer a target for the pharmacological treatment of the metabolic syndrome with altered NPY levels.

## Introduction

The endocannabinoid system comprises lipid mediators known as endocannabinoids, that is, anandamide (*N*-arachidonoylethanolamine, AEA) and 2-arachidonoylglycerol (2-AG), the metabolic enzymes for these two compounds and their molecular targets, that is, the cannabinoid receptors type-1 (CB1) and -2 (CB2).^1^ CB1 receptors are expressed in brain areas controlling food intake and reward, such as the hypothalamus and the mesolimbic system, but also in peripheral organs, such as the white adipose tissue (WAT) and the liver, which have a fundamental role in lipid and glucose biosynthesis, storage and processing.^2, 3^ In these tissues, activation of CB1 receptors by endocannabinoids is strongly dependent on the nutritional status,^4^ and is regulated by hormones and peptides involved in energy homeostasis such as leptin, ghrelin and glucocorticoids.^5, 6, 7^ Furthermore, the physiological activity of the endocannabinoid system becomes deregulated during obesity and related metabolic disorders, thus contributing to the worsening of the latter.^8^ Blockers of CB1 receptors (regardless of whether they are inverse agonists or neutral antagonists) or inhibitors of 2-AG biosynthesis are able to reduce food intake and body weight, and in the case of the former compounds, to counteract the hypertriglyceridemia, glucose intolerance, insulin resistance and dyslipidemia that usually accompany obesity.^1, 9^ The CB1 antagonist rimonabant was previously used as an effective anti-obesity drug, but withdrawn from the market due to adverse effects in the central nervous system.^10^ However, novel compounds acting only on peripheral CB1 receptors are emerging as an attractive therapeutic option for metabolic diseases, even though they do not reduce food intake and are usually less effective at reducing body weight.^1^

Neuropeptide Y (NPY) is a well-established player in the hypothalamic control of body energy balance and one of the key components of the interconnected orexigenic networks upregulated during negative energy balance. NPY in the hypothalamus is a powerful orexigenic factor that induces obesity also by promoting lipid storage, inhibiting brown adipose tissue thermogenesis and inducing hyperinsulinemia and hypercorticosteronemia.^11, 12^ A gain-of-function polymorphism in the *Npy* gene has been associated with traits of the metabolic syndrome (MS), but, paradoxically, not with hyperphagia or obesity.^13, 14^ NPY and its receptors, like the endocannabinoids and CB1, are located in key metabolic tissues, such as the adipose tissue, liver and pancreas, and recent evidence suggests that they have an important role in promoting fat storage and accompanying metabolic disturbances.^15, 16, 17^ The source of peripheral NPY is the sympathetic nervous system, where NPY is a co-transmitter with norepinephrine.^18^ To characterize the role of NPY co-localized with norepinephrine in sympathetic nervous system and brain noradrenergic neurons, we previously created a transgenic mouse model overexpressing NPY under the promoter of the *dopamine–β-hydroxylase (DβH)* gene (OE-NPY^DβH^ mice).^19^ Fitting with the association of the human *Npy* gene variant with the MS and supporting an important role for peripheral and brainstem NPY, the transgenic mice developed an MS-like phenotype. The metabolic disturbances were intensified by increased transgene copy number. Already at the age of 4 months homozygous OE-NPY^DβH^ mice were characterized by 7–8% increase in body weight, 55–74% increase in WAT mass, hypertrophic adipocytes, impaired glucose tolerance and insulin resistance in comparison with WT control mice.^20^ Along with obesity, they also develop hepatosteatosis.^20, 21^ However, food intake and energy expenditure are normal in OE-NPY^DβH^ mice and thus the metabolic phenotype is suggested to result from the direct effects of NPY on peripheral tissues and the downregulation of sympathetic tone.^20^

The endocannabinoid and NPY systems have previously been shown to interact in the hypothalamus. Hypothalamic NPY release is increased by pharmacological stimulation of CB1 receptors and inhibited by CB1 blockage.^22^ However, NPY signaling is necessary for the stimulatory effect of CB1 blockers on corticosterone levels,^23^ but not for their inhibition of food intake.^5, 23^ On the other hand, NPY orexigenic actions require the presence of active CB1 receptors,^24^ suggesting that some of the effects of NPY are mediated by endocannabinoids. The current work aimed at testing the hypothesis that endocannabinoids are also mediating the effects of NPY co-localized with norepinephrine on the development of obesity and MS-like phenotype. For this purpose, we studied whether hypothalamic and peripheral levels of endocannabinoids were altered in 2-, 4- and 7-month-old OE-NPY^DβH^ transgenic mice with a metabolic phenotype described elsewhere^20^ and whether these mice respond to a chronic administration of a CB1 antagonist/inverse agonist with a reduction in body fat, glucose intolerance and liver adiposity.

## Materials and methods

### Animals

Homozygous transgenic OE-NPY^DβH^ and control wild-type (WT) male mice on a C57Bl/6 N background^25^ were produced by homozygous and WT parents that originated from the same heterozygous litters. The mice were genotyped by qPCR as previously described^25^ using transgene-specific primers that resulted in gene expression with a fold change indicating the copy number of the transgene (0, 1, 2 for WT, heterozygous or homozygous mice, respectively). The transgene insertion site in the genome is within the protamine-1 gene that is involved in spermatogenesis,^20^ but does not lead to reproductive disturbances in the homozygous transgenic mice.^25^ The mice were housed in an animal room maintained at 21±3 °C with a 12- h light/12- h dark cycle (lights on at 0600 h). Standard rodent chow (9 kcal% fat, 22 kcal% protein, 69 kcal% carbohydrates, SDS, Essex, UK) and tap water were freely available. At the time of killing, mice were anesthetized with ketamine (75 mg kg^−1^ intraperitoneally (i.p.) Ketalar, Pfizer Oy, Helsinki, Finland) and medetomidine (1 mg kg^−1^ i.p. Domitor, Orion Oyj, Espoo, Finland), and serum was obtained by heart puncture as a terminal blood sample after 4- h fast. The anesthetics were chosen as in our previous work they did not mask the differences between OE-NPY^DβH^ and WT mice in tissue levels of NPY or serum levels of catecholamines,^19, 20, 26^ and as they may not have similar to propofol an inducing effect on endocannabinoid levels.^27^ Animal care was in accordance with the guidelines of the ICLAS (International Council of Laboratory Animal Science), and all experimental procedures were approved by the national animal care and use committee.

### Tissue endocannabinoid levels

Male OE-NPY^DβH^ and WT mice at the age of 2, 4 and 7 months were killed and hypothalamus, epididymal WAT, liver, pancreas and soleus muscle were collected, snap-frozen within 10 min after the death of the animal and stored in −70 °C until the endocannabinoid level measurements. An *n*=4 of mice per group was used as this number was found to be necessary and sufficient to detect statistically significant changes in several previous studies from our and other groups, and to minimize the use of animals. The medial basal hypothalamus was isolated with a mouse brain block using a 3-mm section caudal to the optic nerve chiasma. Lipids were extracted from tissues, endocannabinoids purified from lipid extracts and endocannabinoid levels quantitated with isotope dilution liquid chromatography-atmospheric pressure chemical ionization-mass spectrometry as previously described.^28^

### Chronic CB1-receptor blockage

Male OE-NPY^DβH^ and WT mice (*n*=9–12/group) received daily i.p. injections of 3 mg kg^−1^ CB1-receptor inverse agonist AM251 [N-(Piperidin-1-yl)-5-(4-iodophenyl)-1-(2,4-dichlorophenyl)-4-methyl-1H-pyrazole-3-carboxamide] (Tocris Bioscience, Bristol, UK) or vehicle for 5 weeks. AM251 is able to cross the blood–brain barrier.^29^ It was selected since it has been widely used in previous as well as in recent^30^ studies, thus providing references to our current findings. The dose was selected based on one such study with 3 mg kg^−1^ causing a moderate but significant effect on food intake and fat mass.^31^ AM251 was diluted to vehicle containing DMSO, Tween-80 (Fisher Scientific, Fair Lawn, NJ, USA) and 0.9% NaCl (1:1:18). Drug administration started at the age of 15 weeks and continued until killing at the age of 20 weeks. Mice were habituated to the stress of injections with daily i.p. saline injections 1 week before the drug treatment. Subsequently, OE-NPY^DβH^ and WT mice were divided into two treatment groups that were similar in their initial body weights (OE-NPY^DβH^: vehicle 29.9±0.6 g, AM251 30.3±0.6 g; WT: vehicle 29.3±0.4 g, AM251 29.2±0.5 g). Group-housed mice were weighed daily and food intake per cage was measured weekly. Weekly food intake per cage was divided by the number of animals in each cage and presented as an average daily food intake per animal per cage.

Body composition was measured from conscious mice 1½ week before the drug treatment, at the initiation of the drug treatment and after 2 and 5 weeks of treatment (referred as weeks −1½, 0, 2 and 5) with EchoMRI-700 (Echo Medical Systems LLC, Houston, TX, USA). Each animal was scanned twice and the average values for body fat mass, lean tissue mass, free water and total body water content were calculated from two separate scans.

Glucose tolerance test (GTT) was performed and plasma corticosterone levels measured after 3 weeks of drug treatment. Mice were fasted for 4 h, glucose (5%, wt/vol, 1 g kg^−1^) was administered i.p. and tail vein glucose was measured at 0, 20, 40, 60 and 90 min with a Precision Xtra Glucose Monitoring Device (Abbott Diabetes Care, Abbot Park, IL, USA). Serum samples for corticosterone analyses were collected at the 0-min glucose sampling. Corticosterone levels were measured with a radioimmunoassay kit ImmunoChem Double Antibody Corticosterone (MP Biomedicals, LLC, Orangeburg, NY, USA) according to the manufacturer's instructions.

At the time of killing, subcutaneous, epididymal, retroperitoneal and mesenteric WAT pads and liver were collected and weighed. Liver lipids were isolated, purified and quantified with Serum triglyceride determination kit (TR0100; Sigma, St. Louis, MO, USA) as published before.^19^ Serum non-esterified fatty acids were quantitated with NEFA-HR(2) kit (Wako Diagnostics, Richmond, VA, USA).

### Statistical analyses

The endocannabinoid levels were compared by ANOVA followed by the Bonferroni's *post hoc* test. Body weight development between treatments in each genotype was determined using Generalized Linear Mixed Model and fitted for body weight accounting for individual mice with Toeplitz covariance structure. *P*-values for multiple comparisons were adjusted using the simulation-based method. Other results comparing genotypes were analyzed with an unpaired Student's *t*-test. Logarithmic transformations or non-parametric Mann–Whitney test was used if data were not normally distributed (D'Agostino and Pearson omnibus normality test). Comparisons between different treatments were analyzed with two-way ANOVA and Bonferroni *post hoc* test. GTT and tests with several time points were analyzed with a repeated-measures two-way ANOVA. Areas under the resultant glucose curves were calculated with the trapezoidal method. Statistical analyses were carried out using GraphPad Prism 6.0 (GraphPad Software, San Diego, CA, USA). Generalized Linear Mixed Models were fitted using SAS System for Windows, version 9.0 (SAS Institute Inc., Cary, NC, USA). Data are presented as means±s.e.m. and the results were considered as statistically significant at *P*<0.05.

## Results

### Tissue endocannabinoid levels

**Figure 1:**
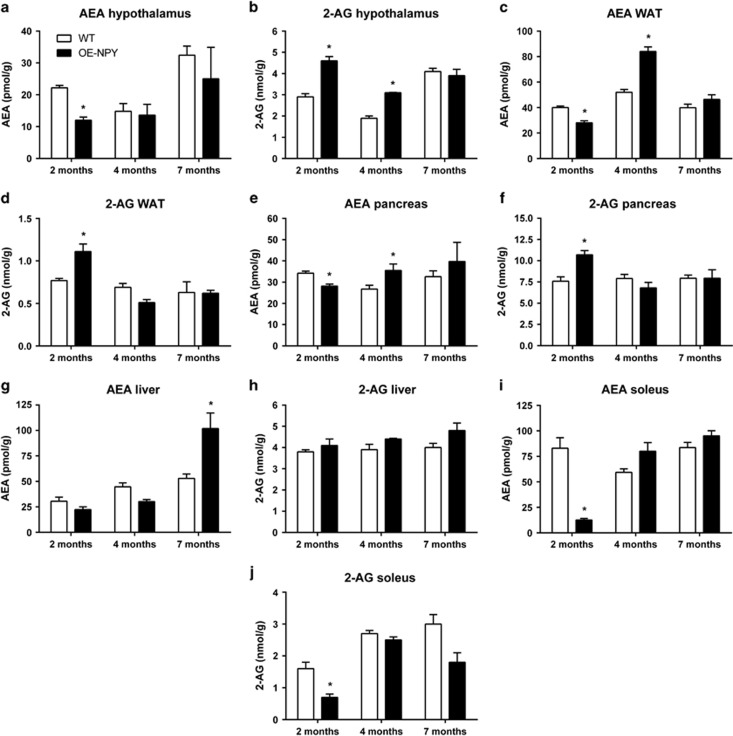
Tissue endocannabinoid levels. AEA and 2-AG levels in the (**a**, **b**) hypothalamus, (**c**, **d**) epididymal WAT, (**e**, **f**) pancreas, (**g**, **h**) liver and (**i**, **j**) soleus muscle of WT and OE-NPY^DβH^ mice. Values are expressed as means±s.e.m., *n*=4. White bars=WT, black bars=OE-NPY^DβH^. *P<0.05 with ANOVA followed by the Bonferroni's test.

Two-month-old OE-NPY^DβH^ mice showed a significant reduction of hypothalamic AEA and elevation of 2-AG levels (Figures 1a and b), although their major metabolic dysfunctions, for example, obesity and impaired glucose tolerance were not yet manifested. At the age of 4 months, OE-NPY^DβH^ mice still had higher hypothalamic 2-AG levels in comparison with WT mice, but not any longer displayed lower AEA levels. No difference was observed in endocannabinoid levels at 7 months between the two genotypes.

In epididymal WAT and pancreas of OE-NPY^DβH^ mice, AEA was significantly reduced and 2-AG elevated at the age of 2 months (Figures 1c and f). However, at the age of 4 months, AEA levels were elevated in OE-NPY^DβH^ mice without changes in 2-AG levels. No difference in endocannabinoid levels between OE-NPY^DβH^ and WT mice was observed at 7 months in either epididymal WAT or pancreas.

In the liver, OE-NPY^DβH^ mice showed elevated AEA levels only at 7 months of age, with no difference between genotypes at a younger age (Figure 1g). The hepatic levels of 2-AG did not differ between the two genotypes at any age (Figure 1h).

The soleus muscle was the only tissue analyzed in which the levels of both endocannabinoids were lower in OE-NPY^DβH^ compared with WT mice at 2 months of age (Figures 1i and j). No difference was observed at 4 or 7 months.

### Chronic CB1-receptor blockage

#### Body weight

**Figure 2:**
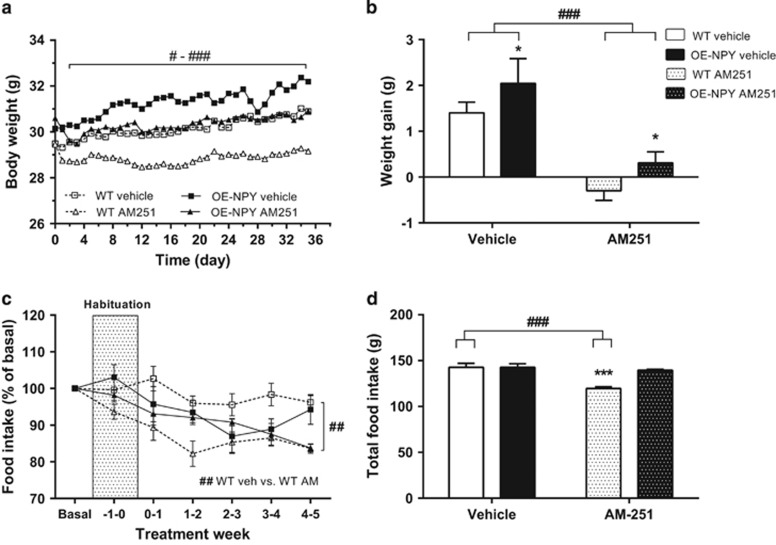
Body weight gain and food intake. (**a**) Body weight development and (**b**) total weight gain during the CB1 inverse agonist treatment period. (**c**) Weekly food intake as a percentage of basal food intake during the habituation and treatment and (**d**) total cumulative food intake during the treatment period. Values are expressed as means±s.e.m., *n*=9–12. ^#^*P*<0.05, ^##^*P*<0.01, ^###^*P*<0.001 comparing different treatments within one genotype. *P<0.05, ***P<0.001 comparing different genotypes within one treatment. Analyzed with (**a**, **c**) repeated measures ANOVA or (**b**, **d**) two-way ANOVA.

AM251 decreased body weight gain significantly in both OE-NPY^DβH^ and WT mice starting from the second day of drug administration while taking into account the baseline weight of individual mice (significance varied between *P*<0.05 and *P*<0.001 when comparing the treatments within one genotype on each day, Figure 2a). Total weight gain over the study period was similarly decreased by AM251 in both genotypes when analyzed with two-way ANOVA (drug effect *P*<0.001, interaction effect *P*=NS, Figure 2b). The genotype effect was also significant, that is, OE-NPY^DβH^ mice showed increased body weight gain in comparison with WT mice. Body weight tended to be increased in OE-NPY^DβH^ mice already before drug administration (OE-NPY^DβH^ 30.4±0.3 g; WT 29.5±0.3 g; *P*=0.05) and became even more significant at the end of the treatment period (*P*=0.006).

#### Food intake

AM251-treatment reduced food intake in WT mice when comparing weekly food intake during treatment period with basal food intake (average food intake over 3 weeks before habituation; Figure 2c). Reduction in food intake was evident also in total food intake during drug administration period (Figure 2d). In OE-NPY^DβH^ mice, food intake was not significantly different between treatments or in comparison with vehicle-treated WT mice.

#### Body composition

**Figure 3:**
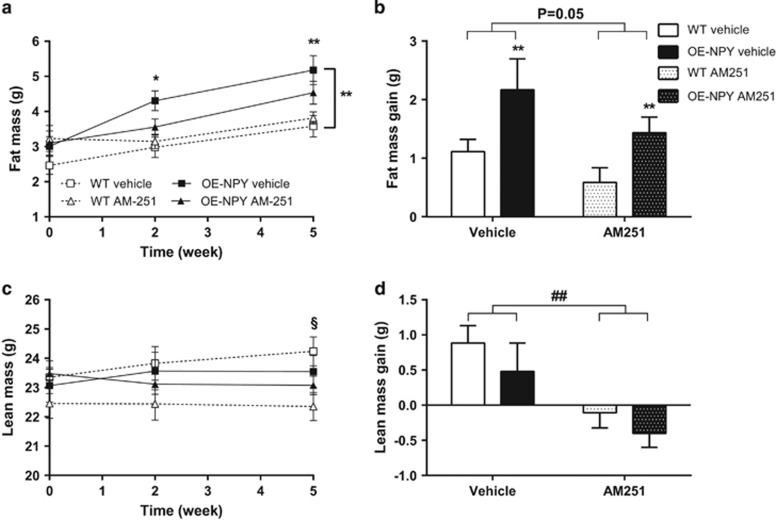
Body composition. Body (**a**) fat and (**c**) lean mass during CB1 inverse agonist treatment period, and total (**b**) fat mass and (**d**) lean mass gain during the whole 5-week treatment period. Values are expressed as means±s.e.m., *n*=9–12. White squares, dashed lines and white bars=vehicle-treated WT; black squares, lines and bars=vehicle-treated OE-NPY^DβH^; white triangles, dashed lines and white dotted bars=AM251-treated WT; black triangles, lines and black dotted bars=AM251-treated OE-NPY^DβH^. *P<0.05, **P<0.01 comparing different genotypes within one treatment, ^##^*P*<0.01 comparing different treatments within one genotype and ^§^*P*<0.05 comparing different treatments only in WT mice with (**a**, **c**) repeated measures ANOVA and *post hoc* test at certain time point or (**b**, **d**) two-way ANOVA.

The genotype difference, that is, OE-NPY^DβH^ mice having more fat in comparison with WT mice in vehicle-treated mice, was significant during the treatment period (Figure 3a). This was evidenced also in the weights of individual WAT pads at the time of killing (vehicle treated: WT 1.41±0.13 g, OE-NPY^DβH^ 1.92±0.15 g; AM251 treated: WT 1.53±0.07 g, OE-NPY^DβH^ 1.91±0.16 g; genotype *P*<0.01, treatment *P*=NS). AM251 showed a strong tendency to suppress fat mass gain in both genotypes (Figure 3b). Lean mass was decreased after 5-week AM251 treatment only in WT mice (Figure 3c) but lean mass gain was decreased after treatment in both genotypes (Figure 3d).

#### Glucose metabolism

**Figure 4:**
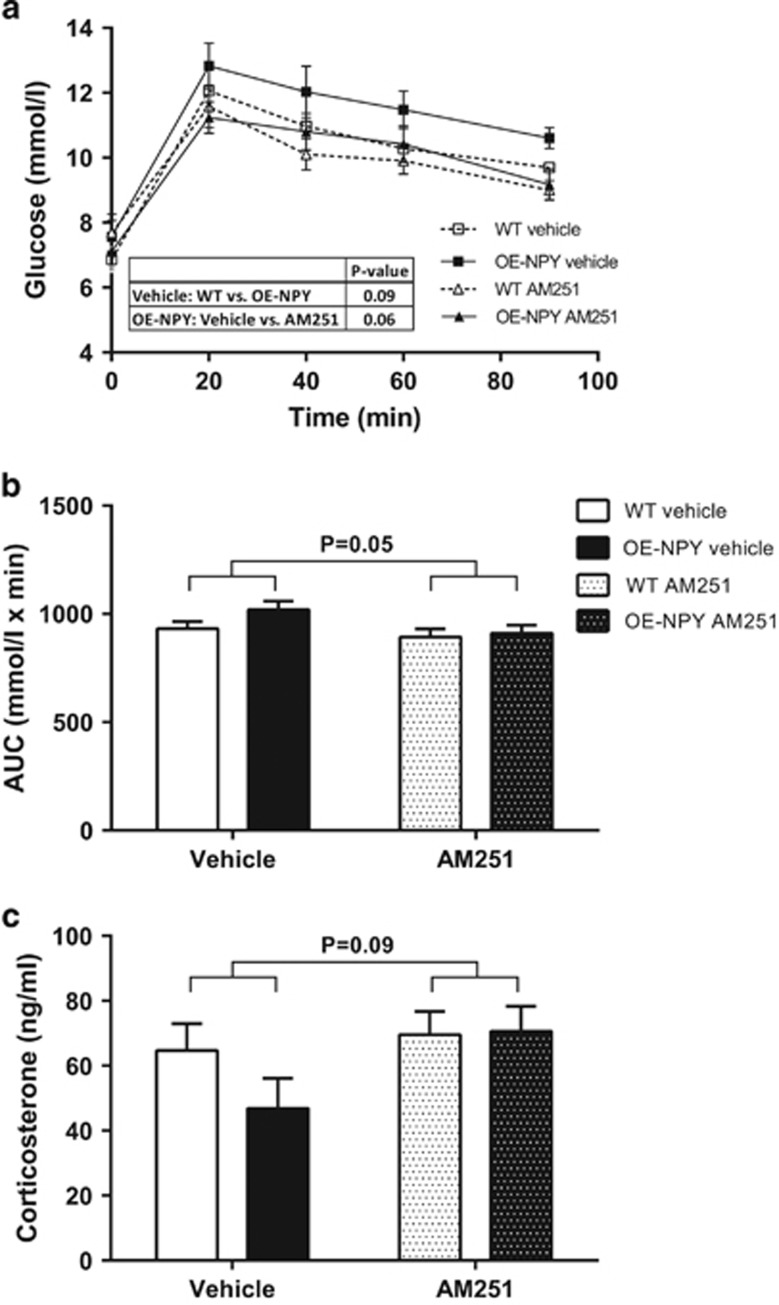
Glucose tolerance and serum corticosterone levels. Glucose tolerance after 3 weeks of CB1 inverse agonist administration as (**a**) blood glucose curves and (**b**) area under the curve values. (**c**) Serum corticosterone levels. Values are expressed as means±s.e.m., *n*=9–12. White square, dashed line and white bars=vehicle-treated WT; black squares, line and bars=vehicle-treated OE-NPY^DβH^; white triangles, dashed line and white dotted bars=AM251-treated WT; black triangles, line and dotted bars=AM251-treated OE-NPY^DβH^. Statistics with (**a**) repeated measures ANOVA or (**b**, **c**) two-way ANOVA.

GTT performed after 3 weeks of drug treatment showed modest differences between groups. Vehicle-treated OE-NPY^DβH^ mice showed a tendency (*P*=0.09) to impaired glucose tolerance in comparison with WT mice. Furthermore, AM251 treatment tended (*P*=0.06) to improve this impairment in the glucose tolerance of OE-NPY^DβH^ mice, but did not affect the glucose tolerance in WT mice (Figure 4a). However, when the AUC values from GTT were compared between all groups by two-way ANOVA, treatment with AM251 tended to improve glucose tolerance in both genotypes (Figure 4b).

#### Serum corticosterone and free fatty acid levels

AM251 tended to increase serum corticosterone levels after 3 weeks of drug treatment (*P*=0.09), but no significant genotype effect was detected (Figure 4c). There were no differences in serum free fatty acid levels between genotypes or treatments after the 5-week treatment period (data not shown).

#### Liver weight and liver adiposity

**Figure 5:**
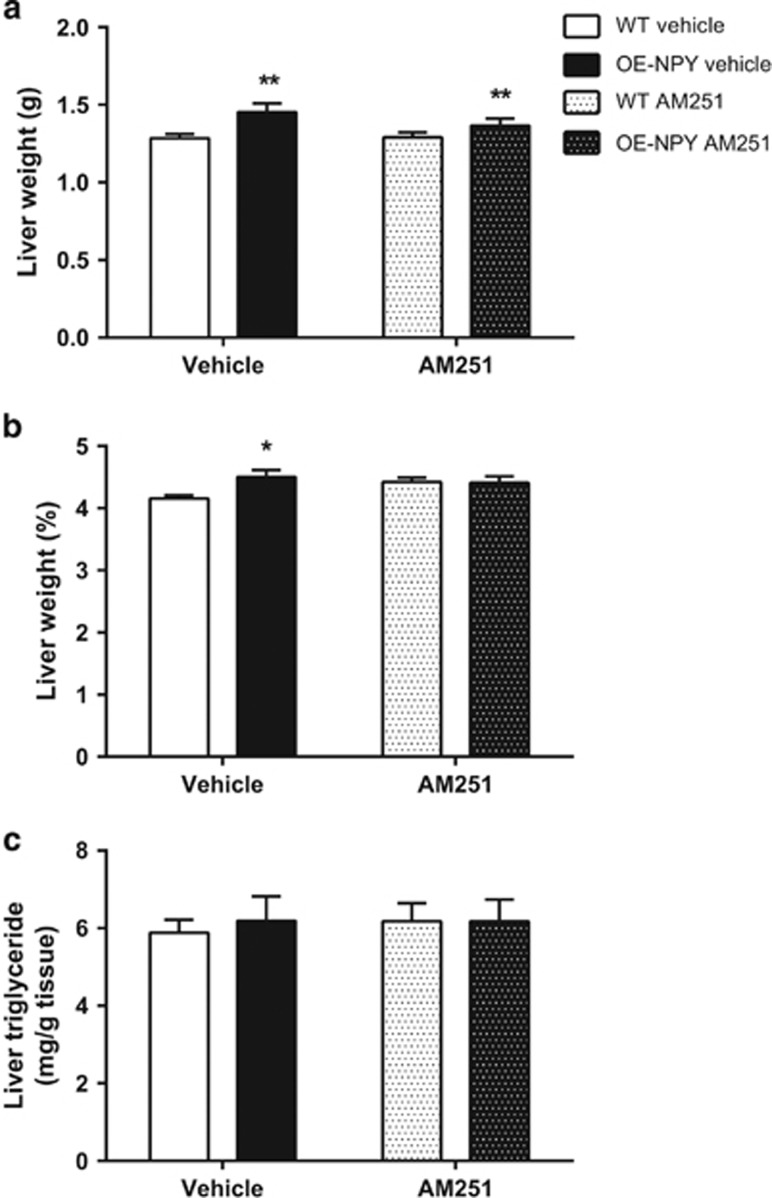
Liver weight and adiposity. Liver weight (**a**) in grams and (**b**) as a percentage of total body weight. (**c**) Liver triglyceride content. Values are expressed as means±s.e.m., *n*=9-12. White bars=vehicle-treated WT; black bars=vehicle-treated OE-NPY^DβH^; white dotted bars=AM251-treated WT; black dotted bars=AM251-treated OE-NPY^DβH^. *P<0.05, **P<0.01 comparing different genotypes within one treatment with two-way ANOVA.

AM251 had no effect on liver weight or liver adiposity. OE-NPY^DβH^ mice showed significantly increased liver weight in grams in comparison with WT mice in both treatment groups (Figure 5a). However, the increase in the liver mass as a percentage of total body weight met significance only in vehicle-treated OE-NPY^DβH^ mice (Figure 5b). There was no difference in liver triglyceride content between genotypes or treatments (Figure 5c).

## Discussion

We report here data suggesting that at least some of the metabolic dysfunctions that are evident in the transgenic OE-NPY^DβH^ mouse model might be due to alterations in endocannabinoid levels in the hypothalamus and peripheral tissues, and subsequent effects on lipid and glucose metabolism. OE-NPY^DβH^ mice develop obesity and metabolic diseases with age.^20, 21, 25^ In young homozygous mice (2 months), increased WAT mass is already evident. Obesity and impaired glucose tolerance develop around 4 months of age, and hepatosteatosis later in life.^20, 21^ The data presented here show that over the course of the development of these metabolic dysfunctions, the levels of endocannabinoids in tissues and organs deputed to lipid and glucose metabolism are also altered. To test whether blocking the endocannabinoid action at the time when endocannabinoids are upregulated changes the phenotype, 4-month-old OE-NPY^DβH^ mice were treated with the CB1 inverse agonist/antagonist AM251. Despite the fact that the daily drug injections compromised weight gain and metabolic disturbances, and might have reduced the magnitude of drug effect, there was a significant attenuation of body weight gain and trends toward the improvement of fat mass gain and glucose tolerance, which normalized the metabolic phenotype of OE-NPY^DβH^ mice to the level of WT mice.

The first trait of the metabolic phenotype in OE-NPY^DβH^ mice is increased adiposity,^20^ which is accompanied by an increase of 2-AG and a decrease of AEA levels in WAT. While these opposing changes could counteract the metabolic effects of each endocannabinoid at an initial stage, the observed higher AEA levels at a later time point (4 months of age) may reinforce the accumulation of triglycerides in this tissue.^32, 33^ This could subsequently contribute to the development of impaired glucose tolerance that has been reported for this same cohort of 4-month-old OE-NPY^DβH^ mice.^20^ In agreement with this finding, OE-NPY^DβH^ mice responded to chronic CB1 blockage by AM251 with trends toward reduced fat mass and improved glucose tolerance. However, there was no statistical difference between genotypes in the response to AM251 treatment. Additionally, lean mass was reduced by AM251 in both genotypes. This effect is not widely reported but is seen also in a previous study by Judge *et al.,*^34^ and might represent an undesired side effect of CB1 receptor blockage.

The hypothalamic levels of endocannabinoids also showed opposing changes in 2-month-old OE-NPY^DβH^ mice, which fits with the lack of hyperphagia.^20^ It is not unusual to find opposing regulation of the brain levels of the two major endocannabinoids, which could be explained by the fact that AEA can inhibit 2-AG biosynthesis in the brain, and 2-AG can act as an alternative substrate for AEA catabolic enzyme, fatty acid amide hydrolase, and be degraded in the place of AEA.^35^ The fact that AEA and 2-AG are not necessarily regulated in the same manner finds a functional raison d'etre in the fact that AEA can act also at non-cannabinoid receptors.^36, 37^ In contrast, 2-AG levels alone were increased in the hypothalamus of 4-month-old OE-NPY^DβH^ mice. This alteration might, therefore, be considered as a secondary effect of leptin resistance^5^ caused by an early onset of excess WAT accumulation rather than one of the direct effects causing the development of obesity in OE-NPY^DβH^ mice. Interestingly, AM251 decreased food intake in WT, but not in OE-NPY^DβH^ mice. This may indicate that the peripheral alterations in endocannabinoid tone of OE-NPY^DβH^ mice are more important for the metabolic effects caused by AM251. This compound, instead, might produce its effects in lean and metabolically healthy WT mice through its inverse agonist properties in the central nervous system (which would not necessarily require enhanced levels of endocannabinoids), rather than by counteracting peripheral endocannabinoid hyperactivity. Previous research has shown that CB1 inverse agonists increase energy expenditure in obese, but not in lean rodents,^38, 39^ which could provide a mechanism for weight reduction in OE-NPY^DβH^ mice. Furthermore, there was a tendency to increased corticosterone levels after AM251 treatment especially in the OE-NPY^DβH^ mice. Higher 2-AG levels in the hypothalamus of OE-NPY^DβH^ mice could tonically inhibit the hypothalamic-pituitary-adrenal axis and CB1-receptor antagonism remove the inhibition and increase circulating corticosterone levels as previously described.^23^ A similar finding has been previously reported for Zucker^fa/fa^ rats,^40^ which also exhibit permanently elevated hypothalamic 2-AG levels.^5^

Increased hypothalamic 2-AG could also mediate the impairment in glucose tolerance in OE-NPY^DβH^ mice. Central infusion of CB1 agonists or elevation of hypothalamic 2-AG levels has been shown to contribute to the hepatic glucose production and insulin resistance.^41^ Fasting hyperinsulinemia developing later in life^20^ could instead be mediated by elevated hepatic endocannabinoid tone (see below), or by the observed elevated pancreatic AEA levels as it has been previously reported that CB1 activation enhances both basal and glucose-induced insulin release from pancreatic β-cells.^32, 42^ Fitting with these findings, chronic treatment with AM251 tended to improve glucose tolerance in OE-NPY^DβH^ more than in WT mice. In contrast, the reduced levels of both AEA and 2-AG in the soleus muscle, although observed only in 2-month-old transgenic mice, are very unlikely to contribute to the glucose intolerance observed in 4-month-old OE-NPY^DβH^ mice as CB1 activation in myotubes is known to inhibit insulin signaling and glucose uptake.^1^ We hypothesize that these early alterations might represent an initial adaptive mechanism to the increased glucose uptake by the skeletal muscle, aimed at counteracting the effect of the early accumulation of WAT, and possibly explaining why glucose intolerance is observed in these mice only some time after this dysfunction, that is, when endocannabinoid levels in the skeletal muscle are no longer different between WT and OE-NPY^DβH^ mice.

Hepatic levels of AEA were increased only in 7-month-old mice, which exhibit hepatosteatosis,^21^ but not in 4-month-old OE-NPY^DβH^ mice, which do not yet overtly present this dysfunction as evidenced in the current study. Although CB1 overactivity in hepatocytes can contribute to both steatosis and hepatic insulin resistance, and AEA level elevation in these cells accompanies both conditions in mice,^43, 44^ our results suggest that increased hepatic AEA may be a consequence rather than a cause of these conditions in OE-NPY^DβH^ mice. Furthermore, chronic CB1 blockage had no effect on liver triglyceride content in OE-NPY^DβH^ mice suggesting that alterations in hepatic endocannabinoid system are not responsible for the triglyceride accumulation to the liver in these mice, whereas they might contribute to sustain over time insulin resistance in these animals. Accordingly, mice receiving AM251 treatment exhibited only minor changes in the liver evidenced by significantly increased liver weight without increase in triglyceride content.

Thus, this study shows that the changes in endocannabinoid levels precede and CB1-receptor blockage attenuates the metabolic alterations in OE-NPY^DβH^ mice. This may suggest that endocannabinoids mediate some of the metabolic disturbances caused by NPY overexpression in noradrenergic neurons. The hypothalamic effect of NPY on food intake has previously been shown to require intact CB1 receptor and is thus an example of endocannabinoids mediating the effects of NPY.^24^ On the other hand, it is possible that CB1 inverse agonism affects energy metabolism also independently from NPY signaling and that the WT-like phenotype observed in OE-NPY^DβH^ mice following treatment with AM251 is the net effect of two opposing systems. This would also explain why CB1 inverse agonism reduced body weight and fat mass gain to the same extent in both genotypes. Previous research describing independent effects of the two systems showed that CB1 antagonism and NPY deficiency had additive effects on body weight gain and lipid oxidation, and that CB1 antagonism reduced food intake independent of NPY.^5, 23^

In conclusion, in this study we described a potential interplay between NPY expressed in noradrenergic neurons and the endocannabinoid system in the control of energy metabolism. It has been observed before that an intact CB1-receptor system is required for the hypothalamic actions of NPY, including the orexigenic effect.^5, 23^ Here we have shown that increased endocannabinoid tone, especially in the adipose tissue and pancreas, precedes the metabolic alterations observed in mice overexpressing NPY in noradrenergic neurons, and that chronic administration of a CB1 inverse agonist normalizes the metabolic phenotype of these mice without reducing food intake. The results suggest that NPY in the periphery and brain noradrenergic neurons may promote obesity and metabolic disease also through modulation of endocannabinoid levels and CB1 receptors. They confirm that CB1 blockers might offer an effective treatment for metabolic disturbances, including those due to overactivity of NPY, also in the absence of effects on food intake, and possibly by acting preferentially in peripheral organs that do not affect this function. This is of relevance to those types of obesity that accompany chronic stress or are due to gain-of-function polymorphisms in the *Npy* gene.
